# Recommendations for the recognition, diagnosis, and management of long COVID: a Delphi study

**DOI:** 10.3399/BJGP.2021.0265

**Published:** 2021-10-05

**Authors:** Martine Nurek, Clare Rayner, Anette Freyer, Sharon Taylor, Linn Järte, Nathalie MacDermott, Brendan C Delaney

**Affiliations:** Department of Surgery and Cancer, Imperial College London, London.; Department of Acute Medicine, Nottingham University Hospitals NHS Trust, Queen’s Medical Centre, Nottingham.; Department of Acute Medicine, Nottingham University Hospitals NHS Trust, Queen’s Medical Centre, Nottingham.; Central and North West London NHS Foundation Trust and honorary senior clinical lecturer, Imperial College School of Medicine, London.; Anaesthetics Department, Morriston Hospital, Swansea Bay University Health Board, Swansea.; King’s College London, St Thomas’ Hospital, London.; Department of Surgery and Cancer, Imperial College London, London, and principal in general practice, Albion Street Group Practice, London.

**Keywords:** clinical guidelines, COVID-19, general practice, long COVID, long-hauler, post-acute sequelae of COVID-19 (PASC), post-COVID-19 condition

## Abstract

**Background:**

In the absence of research into therapies and care pathways for long COVID, guidance based on ‘emerging experience’ is needed.

**Aim:**

To provide a rapid expert guide for GPs and long COVID clinical services.

**Design and setting:**

A Delphi study was conducted with a panel of primary and secondary care doctors.

**Method:**

Recommendations were generated relating to the investigation and management of long COVID. These were distributed online to a panel of UK doctors (any specialty) with an interest in, lived experience of, and/or experience treating long COVID. Over two rounds of Delphi testing, panellists indicated their agreement with each recommendation (using a five-point Likert scale) and provided comments. Recommendations eliciting a response of ‘strongly agree’, ‘agree’, or ‘neither agree nor disagree’ from 90% or more of responders were taken as showing consensus.

**Results:**

Thirty-three clinicians representing 14 specialties reached consensus on 35 recommendations. Chiefly, GPs should consider long COVID in the presence of a wide range of presenting features (not limited to fatigue and breathlessness) and exclude differential diagnoses where appropriate. Detailed history and examination with baseline investigations should be conducted in primary care. Indications for further investigation and specific therapies (for myocarditis, postural tachycardia syndrome, mast cell disorder) include hypoxia/desaturation, chest pain, palpitations, and histamine-related symptoms. Rehabilitation should be individualised, with careful activity pacing (to avoid relapse) and multidisciplinary support.

**Conclusion:**

Long COVID clinics should operate as part of an integrated care system, with GPs playing a key role in the multidisciplinary team. Holistic care pathways, investigation of specific complications, management of potential symptom clusters, and tailored rehabilitation are needed.

## INTRODUCTION

‘Post COVID-19 condition’ is an umbrella term for a complex, multisystem illness that follows on from an acute COVID-19 infection, irrespective of severity, either immediately or some time after apparent recovery. The terms ‘long COVID’ (UK) and ‘long-haulers’ (US) were adopted by patient groups as people came together to compare their experiences.^1^ Much of the publicity and campaigning has been around the term ‘long COVID’; however, the World Health Organization (WHO) and SNOMED International have adopted the term ‘post COVID-19 condition’ for classification. The term ‘long COVID’ is used in this article but the authors accept that medical terminology may continue with ‘post COVID-19 condition’ or ‘post-acute sequelae of SARS-CoV-2’ in the US.

It is estimated that over 1 million people in the UK (population: 66 million) are currently living with long COVID (prolonged symptoms at ≥4 weeks).^2^ No such data are available for other countries, but numbers are likely to be proportionate, placing huge and immediate pressure on health services worldwide. Associated conditions have yet to be fully delineated, but examples are included in Box 1.^3^ The illness commonly has an unpredictable, relapsing–remitting pattern with significant associated conditions often appearing weeks to months into the disease course. A high index of suspicion and a low threshold for referral to secondary care specialists or doctor-led long COVID clinics with diagnostic capabilities, as per local availability, is therefore advised.

**Box 1.** Known examples of conditions associated with long COVID

Myocarditis or pericarditis Microvascular angina Cardiac arrhythmias, including inappropriate sinus tachycardia, atrial flutter, atrial fibrillation, and high burden of ventricular ectopics Dysautonomia, including postural (orthostatic) tachycardia syndrome (PoTS) Mast cell activation, including urticaria, angioedema, and histamine intolerance Interstitial lung disease Thromboembolic disease (for example, pulmonary emboli, microthrombi, or cerebral venous thrombosis) Myelopathy, neuropathy, and neurocognitive disorders Renal impairment New-onset diabetes and thyroiditis Hepatitis and abnormal liver enzymes Persistent gastrointestinal disturbance, including heartburn, diarrhoea, and loss of appetite New-onset allergies and anaphylaxis Dysphonia

In December 2020, the UK National Institute for Health and Care Excellence (NICE) produced a ‘rapid guideline’,^4^ alongside the launch of both community-based and specialist clinics for long COVID. Discussion among an online community of doctors with long COVID (members of the ‘UK doctors #longcovid’ group on Facebook) quickly identified that there was a practical gap between best-consensus practice in clinical care and the cautious, evidence-based approach adopted by NICE (itself limited by the paucity of evidence concerning investigation and treatment of this new condition).^5^ In such particular circumstances, it is necessary to turn to clinical experience during 2020 as a guide for managing long COVID. Clinicians with lived experience of the condition have been instrumental in helping to define the problem, and ‘expert clinician–patient’ self-help groups remain a vital resource in providing advice to the wider clinical community.

**Table:** How this fits in

There is an urgent need to devise clinical pathways and guidance for long COVID, which is thought to affect 10% of those diagnosed with COVID-19. In the absence of conclusive research to inform clinical practice, ‘expert physician–patients’ (that is, doctors with long COVID and those involved in nascent clinics) are a source of professional expertise. Using robust consensus methodology (the Delphi process), 35 clear and practical recommendations were derived to assist in the organisation of clinics, and the diagnosis and management of patients with long COVID. Medically led multidisciplinary clinics are required as serious cardiovascular, neurocognitive, respiratory, and immune sequelae can present with only non-specific symptoms.

To capture, organise, and disseminate this knowledge, robust consensus-based methods to derive recommendations for best practice in the recognition, investigation, and management of long COVID have been used. These recommendations are intended to guide generalist doctors who are providing medical supervision of a community-based long COVID clinic and who have access to specialist referrals if required.

## METHOD

### Panel selection

A Delphi panel should consist of experts in the area of interest. In long COVID, there is developing expertise through experience. Research studies have only recently begun to receive funding, but doctors with long COVID have over the past year carried out a dynamic discussion on social media, highlighting new case reports, important studies, and potential clinical advances. Many of these doctors are involved in research and/or publishing articles. At this point, a pool of doctors with lived experience of long COVID, combined with UK-based clinicians (of varying specialties) involved in service provision for long COVID, form a suitable expert group.^6^

A call for panellists was placed on the ‘UK doctors #longcovid’ support group, hosted by the social media platform Facebook. This is a closed group, exclusive to UK doctors with an interest in long COVID, many of whom are seeing patients with long COVID and/or have lived experience of the condition themselves (currently, approximately 1100 members). Doctors interested in joining the panel were asked to provide their email address by direct message. To ensure representation of all relevant specialties, specialist experts known to the authors were approached directly (via email) and invited to join the panel.

### The Delphi process

The present Delphi study comprised three stages, described below.

#### Initial identification of items

Following the publication of the NICE rapid guideline^4^ and a ‘living review’ by the UK National Institute for Health Research (NIHR),^7^ the named authors of this article generated a list of potential recommendations (‘items’) to cover common clinical problems that were not fully addressed in the NICE guidance. These were refined in a series of Zoom meetings to create 33 statements. Panellists had all seen the NIHR review and NICE guidance, had been following the research literature, and — more importantly in this case — following evolving clinical experience.

#### Round 1

Panellists were emailed a link to an online questionnaire (hosted by Qualtrics) with a request to respond within 4 days. Upon clicking the link, they gave their contact information (name, email address), qualifications (specialty, year of qualification) and consent to participate in the Delphi study (it was made clear that the results would be published in the form of a scientific article authored by the panel as a whole, and that each panellist would have the opportunity to review the article before submission). Thereafter, they read a brief introduction of the study processes and a definition of long COVID (with examples).^3^ They then indicated the degree to which they agreed with each of the 33 initial items (presented sequentially) on a five-point Likert scale (1 = ‘strongly disagree’, 2 = ‘disagree’, 3 = ‘neither agree nor disagree’, 4 = ‘agree’, 5 = ‘strongly agree’). They were also encouraged to comment on each item in a free-text box, particularly if they disagreed with the item. Item scoring and comments were downloaded from Qualtrics (by the first author), anonymised and summarised (by the first author), and circulated to the other named authors to facilitate review and revision of the items.

Items eliciting a response of ‘strongly agree’, ‘agree’, or ‘neither agree nor disagree’ from 90% or more of panellists were taken as showing consensus. Items with consensus were subject to minor amendment for sense only. If more substantial amendments were needed, the item was reworked on the basis of panel comments and re-tested in Round 2, alongside items that did not obtain consensus initially.

#### Round 2

Responders who took part in Round 1 were emailed a link to a second Qualtrics questionnaire (Round 2), with a request to respond within 5 days. As in Round 1, they indicated their level of agreement with sequentially presented items (either amended from Round 1 or newly added to Round 2) using the five-point Likert scale and provided comments. Items that achieved consensus were included in the final list of recommendations (with minor amendments as before to reflect participants’ feedback). Items that failed to achieve consensus were not included in the final list.

## RESULTS

### Panel characteristics

Thirty-seven doctors responded to the Facebook call and were emailed a link to Round 1; of these, 29 (78%) completed it. A further eight clinicians involved in service provision for long COVID (known to the research team) were emailed a link to Round 1; of these, four (50%) completed it and four (50%) were too busy with acute COVID work. All of the doctors who completed Round 1 also completed Round 2 (that is, no doctors were lost to follow-up).

**Figure 1.**
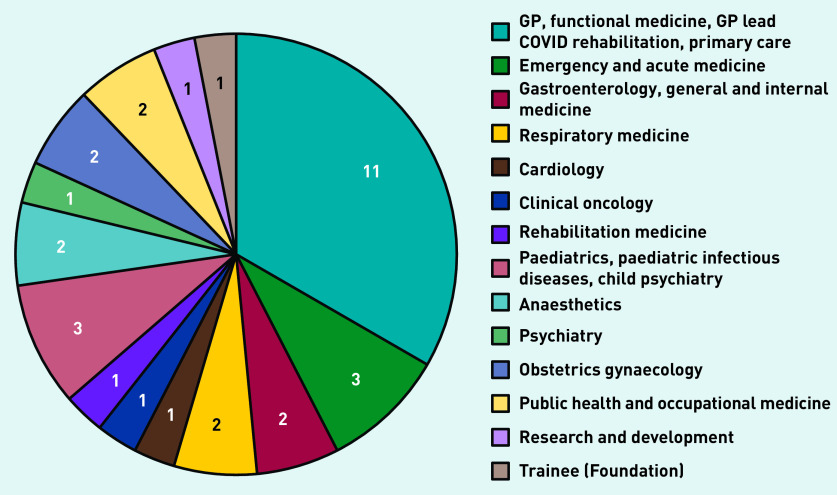
*Number of panellists by specialty.*

The panel therefore comprised 33 UK-based clinicians, representing a wide range of specialties (Figure 1). The median number of years since qualification was 21 (range 0–41). Twenty-nine (88%) were recruited via social media and four (12%) via direct email. Twenty-nine (88%) had lived experience of long COVID and five (15%) were clinicians developing services for long COVID.

### Round 1

Data collection took place over a 9-day period (24 January to 1 February 2021). All 33 items obtained consensus. Of these, 18 required no/minor changes and were incorporated into the final list of recommendations (Supplementary Table S1: Round 1, blue text). The remaining 15 required more substantial work: 13 were amended to reflect panellists’ feedback (Supplementary Table S1: Round 1, green text) and two were excluded (Supplementary Table S1: Round 1, red text). One of the excluded items was deemed self-evident (item 38); the other was strongly opposed by a relevant (respiratory) specialist (item 39).

### Round 2

Round 2 data were collected over a 6-day period (11 February to 16 February 2021). Of the 19 items presented, 13 were amended from Round 1 and six were newly added (usually to disentangle diagnosis and treatment when a Round 1 statement had addressed both). Eighteen items achieved consensus; of these, 17 were added to the final list of recommendations (Supplementary Table S1: Round 2, blue text) and one was excluded (Supplementary Table S1: Round 2, item 36): it was superfluous to another item that had obtained better agreement (agreement for item 36 = 97% versus agreement for item 20 = 100%). One item did not obtain consensus (item 37, agreement = 64%) and the named authors did not feel that there would be a route to achieving this; therefore, this item was also excluded. Supplementary Figure 1 displays the results of the Delphi process graphically.

### Final list of recommendations

The final list comprises 35 recommendations: six relating to clinic organisation (Box 2), 13 to diagnosis of the underlying disorder (Box 3), and 16 to management (Boxes 4 and 5). For a full and printable list of the recommendations, see Supplementary Box S1.^8^^–^^14^

**Box 2.** Recommendations relating to clinic organisation (questions 1 to 6)

Consider long COVID in patients with a clinical diagnosis of COVID-19 as per WHO criteria^8^ or test- positive history with new or fluctuating symptoms including but not limited to breathlessness, chest pain, palpitations, inappropriate tachycardia, wheeze, stridor, urticaria, abdominal pain, diarrhoea, arthralgia, neuralgia, dysphonia, fatigue including neurocognitive fatigue, cognitive impairment, prolonged pyrexia, and neuropathy occurring beyond 4 weeks of initial COVID-19 *(strongly agree 19, 58%; agree 11, 33%; neither agree nor disagree = 0, 0%; disagree = 2, 6%; strongly disagree = 1, 3%).* Multi-specialty long COVID clinics should be led by a doctor with cross-specialty knowledge and experience of managing this condition *(strongly agree = 29, 88%; agree = 2, 6%; neither agree nor disagree = 1, 3%; disagree = 0, 0%; strongly disagree = 1, 3%).* Consider individualised investigations, management, and rehabilitation planning via a multi-specialty long COVID assessment service as local services allow. Prioritise physician-led medical assessments and diagnostics initially, and consider allied health professionals including physiotherapy and occupational therapist input as adjuncts *(strongly agree = 23, 70%; agree = 8, 24%; neither agree nor disagree = 1, 3%; disagree = 1, 3%; strongly disagree = 0, 0%)*. It is inappropriate for long COVID clinics to be led by mental health specialists, for example, IAPT [Improved Access to Psychological Therapy], clinical or health psychologist. They may be useful in supporting the multi-specialty team but do not have the expertise to investigate and manage potential organ damage *(strongly agree = 27, 82%; agree = 5, 15%; neither agree nor disagree = 1, 3%; disagree = 0, 0%; strongly disagree = 0, 0%).* All under-18-year-olds need access to similar services run by paediatric specialists with knowledge of how presentations and treatments differ for adults and with close liaison with school *(strongly agree = 26, 79%; agree = 7, 21%; neither agree nor disagree = 0, 0%; disagree = 0, 0%; strongly disagree = 0, 0%).* Patients with comorbid mental health difficulties should have equal access to medical care as a patient without mental health difficulties and should not be triaged away from services *(strongly agree = 28, 85%; agree = 5, 15%; neither agree nor disagree = 0, 0%; disagree = 0, 0%; strongly disagree = 0, 0%).*

**Box 3.** Recommendations relating to diagnosis of underlying disorders (questions 7 to 19)

**General approach** 7. In someone with long COVID, symptoms of possible non-COVID-19-related issues should be investigated and referred as per local guidelines. Long COVID alone is not a sufficient diagnosis unless other causes have been excluded *(strongly agree = 21, 64%; agree = 8, 24%; neither agree nor disagree = 2, 6%; disagree = 1, 3%; strongly disagree = 1, 3%).* 8. Carry out a face-to-face assessment including a thorough history and examination, consider other non-COVID-19-related diagnoses, and measure full blood count, renal function, C-reactive protein, liver function test, thyroid function, haemoglobin A_1c_ (HbA_1c_), vitamin D, magnesium,^a^ B_12_, folate, ferritin, and bone studies (*strongly agree = 24, 73%; agree = 9, 27%; neither agree nor disagree = 0, 0%; disagree = 0, 0%; strongly disagree = 0, 0%*).
**Respiratory** 9. In those with respiratory symptoms, consider chest X-ray at an early stage. Be aware that a normal appearance does not exclude respiratory pathology *(strongly agree = 27, 82%; agree = 4, 12%; neither agree nor disagree = 1, 3%; disagree = 1, 3%; strongly disagree = 0, 0%).* 10. Be aware that simple spirometry may be normal but patients may have diffusion defects indicative of scarring, chronic pulmonary embolisms, or microthrombi. Consider referral to respiratory for full lung function testing *(strongly agree = 23, 70%; agree = 10, 30%; neither agree nor disagree = 0, 0%; disagree = 0, 0%; strongly disagree = 0, 0%)* 11. Measure oxygen saturation at rest and after an age-appropriate brief exercise test in people with breathlessness and refer for investigation if hypoxaemic or if any desaturation on exercise *(strongly agree = 17, 52%; agree = 14, 42%; neither agree nor disagree = 2, 6%; disagree = 0, 0%; strongly disagree = 0, 0%).*
**Cardiac** 12. Consider the possibility of a cardiac cause of breathlessness *(strongly agree = 27, 82%; agree = 5, 15%; neither agree nor disagree = 0, 0%; disagree = 0, 0%; strongly disagree = 1, 3%).* 13. Be aware that a normal D-dimer may not exclude thromboembolism, especially in a chronic setting, and referral for investigation is therefore indicated if there is a clinical suspicion of pulmonary emboli. Additionally, be mindful that thromboembolism may occur at any stage during the disease course *(strongly agree = 26, 79%; agree = 6, 18%; neither agree nor disagree = 1, 3%; disagree = 0, 0%; strongly disagree = 0, 0%).* 14. In patients with inappropriate tachycardia and/or chest pain, carry out electrocardiogram, troponin, Holter monitoring, and echocardiography. Be aware that myocarditis and pericarditis cannot be excluded on echocardiography alone *(strongly agree = 22, 67%; agree = 8, 24%; neither agree nor disagree = 2, 6%; disagree = 1, 3%; strongly disagree = 0, 0%).* 15. In patients with chest pain, consider a referral to cardiology as cardiac magnetic resonance imaging may be indicated in a normal echo to rule out myopericarditis and microvascular angina *(strongly agree = 25, 76%; agree = 6, 18%; neither agree nor disagree = 1, 3%; disagree = 1, 3%; strongly disagree = 0, 0%).* 16. In patients with palpitations and/or tachycardia, consider autonomic dysfunction *(strongly agree = 25, 76%; agree = 7, 21%; neither agree nor disagree = 0, 0%; disagree = 0, 0%; strongly disagree = 1, 3%).*
**Others** 17. In patients with urticaria, conjunctivitis, wheeze, inappropriate tachycardia, palpitations, shortness of breath, heartburn, abdominal cramps or bloating, diarrhoea, sleep disturbance, or neurocognitive fatigue,^9^ consider mast cell disorder *(strongly agree = 15, 46%; agree = 14, 42%; neither agree nor disagree = 4, 12%; disagree = 0, 0%; strongly disagree = 0, 0%).* 18. In patients with cognitive difficulties sufficient to interfere with work or social functioning, consider neurocognitive assessment *(strongly agree = 23, 70%; agree = 9, 27%; neither agree nor disagree = 0, 0%; disagree = 1, 3%; strongly disagree = 0, 0%).* 19. In patients with joint swelling and arthralgia, consider a diagnosis of reactive arthritis or new connective tissue disease and investigate and refer as appropriate *(strongly agree = 20, 61%; agree = 12, 36%; neither agree nor disagree = 1, 3%; disagree = 0, 0%; strongly disagree = 0, 0%).*

a *Magnesium level may not be available in general practice.*

**Box 4.** Recommendations relating to management: general approach (questions 20 to 27)

20. For patients with fatigue and worsening symptoms hours to days following an activity, emphasise the importance of an initial phase of convalescence followed by careful pacing and rest *(strongly agree = 27, 82%; agree = 6, 18%; neither agree nor disagree = 0, 0%; disagree = 0, 0%; strongly disagree = 0, 0%).* 21. Support patients in shifting their mental timeline of recovery to reflect the likely prolonged course, with a possibly long phased return to work *(strongly agree = 24, 73%; agree = 9, 27%; neither agree nor disagree = 0, 0%; disagree = 0, 0%; strongly disagree = 0, 0%).* 22. Further support patients with signposting to patient resources. Applicable resources may include: management of post-exertional symptom exacerbation, activity pacing, acupuncture, diagnosis-specific management as relevant *(strongly agree = 14, 42%; agree = 16, 49%; neither agree nor disagree = 1, 3%; disagree = 2, 6%; strongly disagree = 0, 0%).* 23. Provide patients with signposting to social prescribing, sickness certification, and financial advice. Discuss with the patient whether sickness certification will state long COVID as diagnosis *(strongly agree = 26, 79%; agree = 6, 18%; neither agree nor disagree = 1, 3%; disagree = 0, 0%; strongly disagree = 0, 0%).* 24. Clinicians should ensure that the occupational status of patients with long COVID is recorded (in/out of work, part-/full-time, student) *(strongly agree = 25, 76%; agree = 8, 24%; neither agree nor disagree = 0, 0%; disagree = 0, 0%; strongly disagree = 0, 0%).* 25. Follow patients up regularly to monitor progress from a full biopsychosocial and occupational perspective *(strongly agree = 19, 58%; agree = 13, 39%; neither agree nor disagree = 1, 3%; disagree = 0, 0%; strongly disagree = 0, 0%).* 26. Encourage reporting of new symptoms (expected) and expectation of waxing–waning course *(strongly agree = 25, 76%; agree = 8, 24%; neither agree nor disagree = 0, 0%; disagree = 0, 0%; strongly disagree = 0, 0%)* 27. Consider contributing patient data to research on long COVID, using the WHO Case Report Form or similar^10^*(strongly agree = 22, 67%; agree = 9, 27%; neither agree nor disagree = 2, 6%; disagree = 0, 0%; strongly disagree = 0, 0%).*

**Box 5.** Recommendations relating to management: specific conditions (questions 28 to 35)

28. Patients with cardiac symptoms should be advised to limit their heart rate to 60% of maximum (usually around 100–110 beats per minute) and investigated with at least electrocardiogram and echocardiogram before taking up exercise. Supervised exercise testing should be considered for this patient group as they may have perimyocarditis and exercise carries risk of arrhythmia and worsening cardiac function^11^ *(strongly agree = 16, 49%; agree = 14, 42%; neither agree nor disagree = 2, 6%; disagree = 1, 3%; strongly disagree = 0, 0%).* 29. For autonomic dysfunction including postural orthostatic tachycardia syndrome (PoTs), consider first increased fluids, salts, compression hosiery, and specific rehabilitation^12^ *(strongly agree = 18, 55%; agree = 13, 39%; neither agree nor disagree = 2, 6%; disagree = 0, 0%; strongly disagree = 0, 0%).* 30. If PoTS and no or inadequate response to non-pharmacological therapy consider beta-blocker, ivabradine, or fludrocortisone (with blood pressure and response monitoring) *(strongly agree = 18, 55%; agree = 13, 39%; neither agree nor disagree = 1, 3%; disagree = 1, 3%; strongly disagree = 0, 0%).* 31. In patients with possible mast cell disorder, consider a 1-month trial of initial medical treatment and dietary advice. Higher than standard dose of antihistamines are commonly used for this indication. If partial effect, consider adding second-level treatment such as montelukast, as well as referral to allergy or immunology specialists^13^^,^^14^ *(strongly agree = 17, 52%; agree = 14, 42%; neither agree nor disagree = 2, 6%; disagree = 0, 0%; strongly disagree = 0, 0%).* 32. Be aware that adverse drug reactions are more common in patients with mast cell disorder, for example, to beta-lactam antibiotics, non-steriodal anti-inflammatory drugs, codeine, morphine, or buprenorphine *(strongly agree = 17, 52%; agree = 13, 39%; neither agree nor disagree = 3, 9%; disagree = 0, 0%; strongly disagree = 0, 0%).* 33. For breathing pattern disorder, consider specialist physiotherapy and/or using alternative therapies such as pranayama breathing and meditation *(strongly agree = 12, 36%; agree = 14, 42%; neither agree nor disagree = 4, 12%; disagree = 3, 9%; strongly disagree = 0, 0%).* 34. In patients expressing distress, significant low mood, anxiety, or symptoms of post-traumatic stress disorder, consider mental health assessment *(strongly agree = 20, 61%; agree = 13, 39%; neither agree nor disagree = 0, 0%; disagree = 0, 0%; strongly disagree = 0, 0%).* 35. Over-the-counter supplementation is common, including vitamin C, D, niacin (nicotinic acid), and quercetin. Be aware of significant drug interactions, such as with niacin or quercetin *(strongly agree = 21, 64%; agree = 10, 30%; neither agree nor disagree = 1, 3%; disagree = 0, 0%; strongly disagree = 1, 3%).*

#### Recommendations relating to clinic organisation (Box 2)

COVID-19 is a new condition with increasing evidence of serious long-term sequelae, including cardiac, respiratory, and renal disease, new-onset diabetes, and excess deaths reported.^15^ It cannot therefore be assumed that patients are suffering from a self-limiting post-viral fatigue and that rehabilitation is sufficient. However, input from expert physiotherapists and occupational therapists who are familiar with the condition is an important aspect of caring for patients with long COVID.^16^^,^^17^ Patients require a holistic clinical approach that prioritises investigation of potential physical pathology.^3^^,^^18^ The lead clinician should be a doctor, *‘well versed in multisystem disorders’*, working across disciplines, and who is able to refer patients to specialists. A responder who runs a long COVID clinic noted the importance of *‘easy access to multi-specialty input without multiple onward referrals (for example, via multi-specialty post COVID MDT* [multidisciplinary team])’ and another that *‘isolated consultant clinics (without full MDT) will not work’*

Long COVID is not a primary mental health problem, but mental health specialists such as neuropsychiatrists can offer a supporting role to the MDT. Psychological aspects of disease should be managed as part of the recovery process, but not seen as the primary treatment focus.^3^^,^^4^ Panellists were clear that no discrimination should exist in the treatment of patients with pre-existing mental health difficulties with regard to equal access to care for their long COVID and appropriate investigations for organ damage.^18^ Regarding children, a consultant paediatrician should lead the service.^3^ NICE guidelines recommend considering referral from 4 weeks for specialist advice for children with ongoing symptomatic COVID- 19 or long COVID.^4^

#### Recommendations relating to diagnosis of underlying disorder (Box 3)

At present there is a considerable risk to patient safety if appropriate investigation of common symptoms of long COVID (such as chest pain, breathlessness, palpitations, abdominal pain, fatigue) that have wide differential diagnoses is not undertaken. Serious conditions, related to severe acute respiratory distress syndrome coronavirus 2 (SARS-CoV-2) infection or not, must be adequately excluded^19^ and investigations should be appropriately guided by the history. Long COVID-specific examination (for example, the NASA Lean Test for postural tachycardia syndrome [PoTS]) or tests such as electrocardiogram are best conducted in person, and chest X-ray (CXR) may be appropriate. CXR may exclude relevant pathology such as tuberculosis but is less relevant in investigating cardiac, pulmonary vascular, or autonomic causes for breathlessness where computed tomography or ventilation/perfusion (V/Q) scans are more likely to be indicated.^20^ In keeping with NICE guidance on asthma management,^21^ the panel agreed that spirometry with beta-agonist reversibility could be used to diagnose airway hyperreactivity.

Studies on venous thromboembolic disease are limited to patients with acute COVID-19,^22^ severe disease,^23^^,^^24^ or based on expert opinion, but are an important diagnosis to consider.^25^ Oxygen desaturation on exertion occurs in both acute and long COVID, and should form part of the baseline assessment. The only thresholds for defining levels of concern for hypoxaemia and desaturation with exercise relate to acute COVID-19,^26^^,^^27^ and no agreed thresholds are available in long COVID. Doctors working in existing clinics indicated that assessments such as 1-minute sit-to-stand tests^28^ and 6-minute walk tests^29^ do or should form part of the assessment in community or specialist clinics. The exertional test chosen should take account of any pre-COVID-19 limitations and should include heart rate as this may help to assess autonomic function. Referral for more detailed assessment is required in the following scenarios: desaturation with or without overt/reported dyspnoea; nocturnal desaturation; extreme fatigue; behavioural change in those who struggle with verbal communication; patient reports significant post-exercise malaise after such testing (lasting beyond the next day); severe tachycardia; postural blood pressure drop.

There is increasing evidence of cardiovascular complications with COVID-19.^30^^–^^37^ Patients with long COVID (of all ages) have been diagnosed with arrhythmias, autonomic dysfunction, myocarditis, pericarditis, and microvascular ischaemia.^38^ The latter three may only be seen on cardiac magnetic resonance (MRI) scans (gadolinium-enhanced; stress). Echocardiography has a low diagnostic yield for myocarditis in long COVID,^38^^,^^39^ but diagnosis is important as people experience significant improvement in daily function through specific treatment. Pulmonary embolism appears to be rare more than 6 weeks after the acute illness and there are feasibility concerns about a potential surge of investigations for long COVID. Usual risk-scoring calculators are not valid in this context^40^ and research is needed.

Autonomic dysfunction, especially manifesting as PoTS, occurs commonly post COVID-19.^12^ There is a need to consider a differential for tachycardia and palpitations that, in long COVID, includes pulmonary embolus, cardiac, and respiratory causes. It was noted that autonomic dysfunction should also be suspected in patients with light-headedness, chest pain, and nausea, and the association of autonomic dysfunction with mast cell disorders considered.

As there are no accepted UK criteria for the diagnosis of ‘mast cell activation syndrome’ and it remains an area of controversy, in the present study the term ‘mast cell disorder’ is used to describe patients who present with a range of features listed in recommendation 17 (Box 3).^41^ The list is not exhaustive and other serious disorders need to be excluded. This area is an important target of mechanistic and potential therapeutic studies in long COVID. Neurocognitive testing is a particularly scarce resource^4^ and neurology review and brain MRI may be more helpful early in the illness. The benefits of testing would support the need for rehabilitation from occupational therapy or a neuropsychologist.^4^^,^^42^ NICE guidelines on long COVID advise considering neuropsychometric testing after 6 months if no improvement/worsening of cognitive function, as many will resolve.^43^ At present, evidence related to joint swelling and arthralgia consists of only case reports, but clinicians should include long COVID in a differential diagnosis of arthritis once other known autoimmune causes have been excluded.^44^

#### Recommendations relating to management (Box 4 and 5)

The experience of many patients is of post-exertional symptom relapse. Physical or cognitive workload beyond the patient’s ‘energy envelope’ may cause an exacerbation of symptoms including fatigue, fever, myalgia, and breathlessness.^45^ Exacerbations may manifest immediately or after a delay of 24–48 hours and may last days or months. As the threshold for this effect varies not only by patient but, over time, pacing needs to be flexible and careful. Doctors play a key role in supporting patients through the complexity of specialist investigations and differential diagnoses, and considering symptomatic treatments. In addition, occupational health service referrals and medical reports supporting the return-towork process are needed.

Employers should discuss with their employee suitable adjustments to aid a return to work, and both parties should be provided with written advice such as the leaflet *COVID-19 Return to Work Guide for Recovering Workers* by the Society of Occupational Medicine.^46^ The relapsing–remitting nature of the illness needs to be emphasised as employer pressure may result in patients returning to work too soon. The onus is on the doctor with current clinical responsibility for the patient to complete the fit note; this includes secondary care doctors.^47^ The content of the fit note should be agreed between the patient and doctor, including a ‘medically recognised diagnosis’. For NHS staff to receive ‘COVID pay’ during absence, the fit note must mention COVID.^48^ The ability to return to work after illness is a marker of recovery and clinicians must, therefore, record work status in the clinical notes in situations of chronic ill health.^49^^,^^50^ From a public health perspective, counting days lost to sickness and lost income on account of long COVID is essential.

Long COVID, like all long-term conditions, has an impact on many aspects of life and is best managed holistically with physical, psychological, and social factors addressed.^51^ Prolonged illness following SARS-CoV-2 infection is characterised by the development of new symptoms at different timepoints.^52^ Clinicians need to provide patient ‘safety-netting’ advice and guidance on expected patterns of illness. Although NICE guidance states that new symptoms after 3 months are unlikely to be because of COVID,^4^ this is not borne out by research^53^ or patient and specialist experience. The importance of research into long COVID was emphasised by many, which should include quantification of the burden of disease. To this end, one panellist advised that case reporting should be mandatory. The length of the WHO long COVID Case Report Form was noted, however, and flagged as a potential barrier to case reporting in practice. Patients should be made aware of research studies: participation could add meaning to what is often a very negative experience.

Ongoing exertional chest pain may warrant referral to a Rapid Access Chest Pain Clinic and/or cardiac MRI.^54^ In patients diagnosed with myocarditis, exercise to 60% maximum heart rate can be advised but patients need to work out their own limits, which may be lower than this. In some cases of myocarditis or pericarditis, there is a difficulty in managing tachycardia and pharmacological approaches are needed, such as beta-blockers or ivabradine. As colchicine and anti-anginals may also be helpful, advice from a long COVID assessment clinic needs to be sought.^15^

PoTS (and other dysautonomic symptoms such as breathlessness, orthostatic intolerance, dizziness, and tremor) is an unfamiliar diagnosis for many clinicians, but seems to affect a significant subgroup of patients with long COVID. Although many would advocate specific investigation, NHS autonomic services are patchy, and, if they are not to be overwhelmed, there will be significant educational needs for referring clinicians.^55^ PoTS treatment can start with fluids, compression, and lifestyle adaptations (for which specific patient support materials are available),^56^^,^^57^ but may need to escalate to medication if symptoms are not improved.^58^^,^^59^ Midodrine may be helpful, although this is only available following secondary care initiating the prescription in many parts of the UK. There is an urgent need for research, education, and clear guidance to help GPs in managing this condition.

Similarly to treating urticaria, mast cell features require two- to fourfold larger doses of antihistamines to suppress them.^9^ Dermatologists and GPs with an interest in mast cell disorders have experience in counselling patients about such off-label use and an individual therapeutic trial is simple to arrange. Some patients exhibit sensitivity to histamine-rich foods and prominent gastrointestinal symptoms (bloating, cramping pain, diarrhoea, acid reflux). These and other known triggers of mast cell activation should be avoided; the aim is to switch off the immune overreaction.^60^ Unfortunately, H_2_-receptor antagonists are not readily available in the UK, although at the time of writing famotidine is again available. Further research including clinical trials are needed in this area, but the recommendations represent a simple solution to dealing with very troublesome symptoms in some patients with long COVID.^15^

The term ‘breathing pattern disorder’ was used in the present study to describe the subjective experience of patients that is not ‘breathlessness’ in the strict sense of the word.^61^ Its aetiology is unknown but may represent a disorder of central breathing control. Although specialist physiotherapy should be available to patients being seen in clinics, many patients seek help from alternative therapy, such as pranayama breathing. Meditation/mindfulness is promoted in the NHS as an effective therapy for anxiety and the sensation of breathlessness.^62^

It has been long established that chronic physical diseases have an increased risk of secondary mental health problems. A meta-analysis showed that 36.6% of people with a chronic physical disease had a coexistent mental health disorder.^63^ Having a mental health disorder should not preclude investigation of any organic disease and unexplained symptoms or signs, and neuropsychiatric features should always prompt exclusion of organic pathology in the first place. Addressing epistemic injustice issues in the investigation and management of long COVID should be a priority for local services.^64^

Patients with long COVID commonly refer to taking ‘the stack’ or ‘the supplement stack’, which includes high-dose vitamin C and D, niacin (nicotinic acid), quercetin, zinc, selenium, and sometimes also magnesium.^65^^–^^67^ Further research is needed to confirm or refute the impact of supplements in long COVID.^68^ Examples of noteworthy interactions with supplements include: niacin causing an increased risk of bleeding events when combined with selective serotonin reuptake inhibitors or non-steroidal anti-inflammatory drugs, increased risk of rhabdomyolysis together with statins,^69^ and quercetin causing inhibition and induction of various human cytochrome P450 enzymes.^70^

A recommendation concerning the name (‘long COVID’) did not obtain consensus and was ultimately excluded (Supplementary Table 1: item 37). In Round 1, the named authors suggested the term ‘long COVID’ in preference to ‘Post COVID-19 Syndrome’ and this achieved consensus (94% agreement); in Round 2, the named authors decided to use the recently approved WHO term ‘Post COVID-19 Condition’ but this did not obtain consensus (64% agreement). The naming of the condition is a subject of considerable controversy:^71^ ‘long COVID’ and ‘long-hauler’ have been adopted by patients in the UK and US, respectively, as neutral terms that make no assumptions about aetiology, presence/absence of ongoing infection, or prognosis.^4^ NICE suggested adoption of two terms: ‘Ongoing Symptomatic COVID-19’ (4–12 weeks) and ‘Post COVID-19 Syndrome’ (12+ weeks).^4^ In the US the term ‘Post-Acute Sequelae of COVID-19’ (PASC) has been adopted.^72^ In contrast, the WHO has adopted ‘Post COVID-19 Condition’ for ICD-11, reflected also in SNOMED coding.^73^ This enables the creation of subclassifications or SNOMED coordinated terms for any future subcategories of long COVID. Given the desire for international adoption and its use in coding, the authors of the present article accept the use of ‘Post COVID-19 Condition’ as another medical term to describe long COVID.

## DISCUSSION

### Summary

With a novel viral pathogen giving rise to a newly recognised condition and a worldwide pandemic, there is at present very little empirical evidence on which to base recommendations for clinical care. Mechanisms that aim to identify, appraise, and use evidence in guidelines cannot function effectively when there is little evidence, and often only hypothecated comparisons with other conditions. In this situation emerging clinical experience is the best practical guide to both practice and research. Cognisant of this, the present study used a robust Delphi method to derive 35 clear and practical recommendations to assist in the recognition, diagnosis, and management of patients with long COVID.

### Strengths and limitations

Recognising the need for strong consensus in the absence of evidence, a high threshold for agreement (90%) was chosen. The midpoint of the Likert scale (‘neither agree nor disagree’) was interpreted as ‘agreement’, but classifying this as ‘disagreement’ would affect only three recommendations (7, 17, and 33), whose consensus would fall below 90% but nonetheless clear a threshold of 75%.^74^ A rich set of views and relevant literature surrounding the recommendations has been summarised, even when a clear consensus was present.

Other than process, the key question for the validity of a Delphi study is the composition of the expert panel. A panel of active ‘clinician–patients’, augmented with clinicians involved in newly established long COVID clinics, was therefore recruited. A wide range of specialisms were represented in the panel and all panellists completed both rounds of the Delphi. This ensures that the recommendations were derived by clinicians highly active in this developing field rather than simply appraising an as-yet sparse literature. The recommendations were written in the spirit of promoting patient safety by ensuring differential diagnosis and the potential for serious underlying conditions are considered. This is the precautionary principle. The authors accept that the full range of experience of long COVID in the community is not yet known and that many patients will recover spontaneously without a need for treatment or referral. However, it is also quite possible that a lack of provision has failed to meet need, and many will have undiagnosed pathology.

### Comparison with existing literature

The statements go further than NICE in many areas, particularly in the need to investigate potential cardiac conditions, dysautonomia, and immune dysfunction. The recently published NIHR *Living with Covid19 — Second Review* echoes many of the themes explored here, including potential subgroups, need for investigation, and the relapsing–remitting nature of the condition, but offers a broad narrative rather than specific, practical statements for clinical use.^75^ Practical management of such issues as PoTS and mast cell dysfunction (which are part of the therapeutic approach to chronic fatigue in the US) can be very helpful for many patients with long COVID and need to be debated and tried in therapeutic settings, eventually as part of controlled studies.

### Implications for research and practice

GPs need to be able to give practical advice to patients with long COVID, especially considering the simple nature of many treatments (for example, compression stockings, fluids, and electrolytes for PoTS; over-the-counter antihistamines for mast cell suppression; pranayama breathing, meditation, and respiratory physiotherapy for breathing pattern disorder). It is hoped that these statements will act as a stimulus for research, medical education, and discussions around services for long COVID.

Research needs to prioritise rapid learning from long COVID clinics, with mixed-methods improvement science, learning from best practice by sharing data, and by targeted mechanistic studies leading eventually to an evidence-based guideline on patient investigation, segmentation, and specific therapies. It is acknowledged that there are significant challenges in resourcing services for long COVID and that strict adherence to these recommendations will not always be possible but the needs of this large group of high-need patients must be met.

NHS England recently announced further funding for long COVID clinics (there are no dedicated services in the other three UK nations). In due course, services for investigation, advice, and rehabilitation should be available to all practices. At present, however, the sheer practicalities of managing hundreds of thousands of patients after two waves of COVID-19 mean that GPs need to develop confidence in good practice for patients with long COVID, basic investigations, deciding on need for referral or investigation, differential diagnosis, safety netting, empathy, and support. These statements are a carefully considered and reasonable approach to helping patients until further evidence is available, generated by a robust consensus method from a unique group of ‘lived-experience’ professionals and front-line clinicians in the field.
